# A Novel Hybrid CNN-SVR for CRISPR/Cas9 Guide RNA Activity Prediction

**DOI:** 10.3389/fgene.2019.01303

**Published:** 2020-01-08

**Authors:** Guishan Zhang, Zhiming Dai, Xianhua Dai

**Affiliations:** ^1^School of Electronics and Information Technology, Sun Yat-sen University, Guangzhou, China; ^2^School of Data and Computer Science, Sun Yat-sen University, Guangzhou, China; ^3^Guangdong Province Key Laboratory of Big Data Analysis and Processing, Sun Yat-sen University, Guangzhou, China; ^4^Southern Marine Science and Engineering Guangdong Laboratory, Zhuhai, China

**Keywords:** CRISPR/Cas9, guide RNA, convolutional neural network, on-target, support vector regression

## Abstract

Accurate prediction of guide RNA (gRNA) on-target efficacy is critical for effective application of CRISPR/Cas9 system. Although some machine learning-based and convolutional neural network (CNN)-based methods have been proposed, prediction accuracy remains to be improved. Here, firstly we improved architectures of current CNNs for predicting gRNA on-target efficacy. Secondly, we proposed a novel hybrid system which combines our improved CNN with support vector regression (SVR). This CNN-SVR system is composed of two major components: a merged CNN as the front-end for extracting gRNA feature and an SVR as the back-end for regression and predicting gRNA cleavage efficiency. We demonstrate that CNN-SVR can effectively exploit features interactions from feed-forward directions to learn deeper features of gRNAs and their corresponding epigenetic features. Experiments on commonly used datasets show that our CNN-SVR system outperforms available state-of-the-art methods in terms of prediction accuracy, generalization, and robustness. Source codes are available at https://github.com/Peppags/CNN-SVR.

## Introduction

The CRISPR/Cas9 system, adapted from a bacterial defense mechanism, is a promising genomic editing tool that has recently revolutionized the field of biology, biotechnology, and medicine (Barrangou et al., 2007). This system consists of a nuclease activity-carrying Cas9 protein and the specificity-programming single guide RNA (gRNA), and the latter of which targets the complex to a genomic region flanked by a protospacer adjacent motif (PAM) (Jinek et al., 2012). Though the CRISPR/Cas9 system is considered to be very specific to perform the preconcerted cleavage on genome, numerous studies have indicated that Cas9 complex also binds to other unintended genomic sites, termed as off-target (Pattanayak et al., 2013; Doench et al., 2016). Thus, design of a gRNA with high on-target efficacy and low off-target effects is an important issue in CRISPR/Cas9 system. It has been shown that on-target activity is partly determined by gRNA intrinsic sequence and chromatin structure of target genomic region, but the underlying molecular mechanism is still not fully understood. Accurate prediction of gRNA on-target activity facilitates maximization of on-target efficacy and minimization of off-target effects, further contributing to the success application of CRISPR/Cas9 system (Hsu et al., 2013; Doench et al., 2014; Xu et al., 2015; Chuai et al., 2016; Doench et al., 2016).

Previous efforts have been made to assist gRNA on-target identification and efficacy prediction based on different design rules. The alignment-based methods align the gRNAs from the given genome purely by locating the PAM [e.g. CCTop (Stemmer et al., 2015)]. Hypothesis driven-based tools empirically score the gRNA efficacy by incorporating the effect of genomic context factors [i.e. CFD (Doench et al., 2016)]. Machine learning-based methods predict the cleavage propensity of a genomic site for a given gRNA by considering different nucleotide features, such as position specific nucleotides and dinucleotides (Doench et al., 2014), GC content (Chari et al., 2015) as well as non-sequence features including thermodynamic stability of gRNA (Doench et al., 2014), amino acid cut position (Chen et al., 2017), and chromatin accessibility (Hinz et al., 2015; Horlbeck et al., 2016; Listgarten et al., 2018). For example, support vector machine (SVM)-based sgRNA Designer found that the position of the target site relative to the transcription start site and position within the protein are the most important factors for gRNA activity prediction (Doench et al., 2016). L1-regularized linear regression-based SSC reported that DNA sequence composition incorporating the preference for cytosine at the cleavage site improved the performance of gRNA on-target prediction (Xu et al., 2015). WU-CRISPR combined sequence and structural features of the gRNA to identify highly active gRNA (Wong et al., 2015). In general, no single feature but rather a combination of feature interactions governs gRNA cleavage efficacy (Wilson et al., 2018). Sophisticated models considering the interactions between the individual features achieved better performance (Aach et al., 2014; Erard et al., 2017). Nevertheless, some correlated features may result in the redundancy (Abadi et al., 2017), further rendering poor prediction outcome. Moreover, the outcomes of machine learning-based tools mainly depend on laborious manual feature engineering. They require considerable domain expertise to design the feature extractor (LeCun et al., 2015).

Deep learning allows computational models that consist of multiple processing layers to learn representations of features with multiple levels of abstraction (LeCun et al., 2015). The layers of features are learned from data by a general-purpose learning procedure instead of human engineers. Recently, several successful deep learning-based models have been provided for predicting CRISPR gRNA on-target activity. For example, Kim et al. proposed Seq-deepCpf1, which used convolutional neural networks (CNNs) to learn the nucleotide features of CRISPR gRNA, and it outperformed previous machine learning algorithms (Kim et al., 2018). Chuai et al. proposed DeepCRISPR that used deep convolutionary denosing neural network-based autoencoder to extract the CRISPR/Cas9 gRNA sequence representation and utilized the fully CNN model to predict the gRNA efficacy (Chuai et al., 2018). Extensive numerical experiments demonstrated DeepCRISPR surpassed the state-of-the-art tools across a variety of human datasets.

The above two CNN-based models showed good performance in CRISPR gRNA efficacy prediction compared with machine learning-based methods. CNNs are multi-layer architectures where the successive layers are designed to learn progressively higher-level features, until the last layer which produces the classifiers (Huang and LeCun, 2006). The last layer of CNN can be considered as a linear classifier operator on feature representation extracted by previous layers. CNN performs well in automatically learning nonlinearity features. However, CNN is not always an optimal choice for classification because the MLP layer following the feature extraction layer contains many trainable parameters. On the contrary, SVM with fixed kernel function has good utility on minimizing generalization error bound when applied to well-behaved feature vectors. Inspired by this, it is interesting to explore the hybrid CNN-SVM system in which CNN is trained to extract features and SVM computes a classifier function in the learned high dimensional feature spaces. To date, CNN-SVM models have shown impressive performance in a wide range of applications, such as object categorization (Huang and LeCun, 2006) and image recognition (Mori et al., 2005; Niu and Suen, 2012). For example, Niu et al. put forward a CNN-SVM model for handwritten digitals recognition with recognition rate of 99.81%. In their work, the proposed CNN-SVM replaced the back propagation neural network classifier with SVM in the last layer of the CNN model (Niu and Suen, 2012). Mori et al. trained a convolutional spiking neural network using different fragment images. The outputs of each layer in the model were input to the SVM model. A 100% face recognition rate was obtained for 600 images of 20 people (Mori et al., 2005). In terms of regression problem, Li et al. proposed CNN combined with support vector regression (CNN-SVR) for no-reference image quality assessment. This method achieved advanced outstanding performance compared with traditional CNN model (Li et al., 2016).

The prior success of CNN-SVM in computer vision inspired us to extend CNN-SVM application to CRISPR/Cas9 gRNA efficacy prediction. Until now, to the best of our knowledge, there is no such application. Previous studies have suggested that CRISPR gRNA efficacy prediction using linear regression achieved better performance than classification (Moreno-Mateos et al., 2015; Kim et al., 2018). Therefore, SVR, which is a common application form of SVM for regression, may be more appropriate for gRNA efficacy prediction when applied to well-behaved feature vectors. In this work, we developed a hybrid architecture incorporating CNN and SVR for CRISPR/Cas9 gRNA on-target activity prediction. The key idea of our system is to train a specialized CNN to extract robust gRNA sequence and epigenetic features, and to provide them to the SVR classifier for predicting gRNA cleavage efficacy. First, we trained the CNN model with back-propagation on the benchmark dataset, aiming at model selection and parameters tuning. Second, the initial CNN features were input into the SVR for training and evaluating. A two-step strategy was performed to select the important features from well-trained CNN intrinsic gradients features. Third, the well-trained CNN-SVR was used to test the independent cell-line dataset. Specifically, the test data was input to the well-trained CNN model to obtain the test features. Using the test feature vector, the well-trained SVR classifier was performed to predict the gRNA cleavage efficacy. Experiments showed improved performance of the proposed CNN-SVR model for CRISPR/Cas9 gRNA on-target activity prediction compared with state-of-the-art algorithms.

## Materials and Methods

### Data Resources

#### Benchmark Dataset

Previous studies have shown that PAM-distal region has a high tolerance for sequence mismatches (Kim et al., 2016; Kleinstiver et al., 2016). To be specific, gRNAs with two mismatches in the first two positions from the 5’ end has little influence on cleavage efficiency (Doench et al., 2014; Doench et al., 2016). Inspired by these studies, Chuai et al. applied a data augmentation procedure by changing each gRNA into a new one with two mismatches in the PAM distal region (Chuai et al., 2018). Consequently, a 23-nt gRNA sequence can be expanded into 16 gRNAs with identical cleavage efficacy. The augmented dataset was generated from ~15,000 gRNAs with known on-target cleavage efficacy. By adopting this data augmentation strategy, they obtained 180512 non-redundant gRNAs. Each observation in the data contains a 23-nt gRNA sequence and its corresponding cleavage efficiency. In this work, we used this augmented dataset as the benchmark data for model selection and pre-training.

#### Four Cell Line Independent Test Datasets

In order to evaluate the performance of our method, we used four public experimental validated gRNA on-target cleavage efficacy independent human datasets, which were integrated and processed by Chuai et al (Chuai et al., 2018). These experimented-based datasets were originally collected from public datasets (Wang et al., 2014; Hart et al., 2015; Doench et al., 2016). They covered gRNAs targeting 1071 genes from four different cell lines, including HCT116 (4239 samples) (Hart et al., 2015), HEK293T (2333 samples) (Doench et al., 2016), HELA (8101 samples) (Hart et al., 2015), and HL60 (2076 samples) (Wang et al., 2014) with redundancy removed. The gRNA on-target activity was strictly restricted to experimental assay, where the cleavage efficiency was defined as the log-fold change in the measured knockout efficacy. Readouts of cleavage efficacies without in vivo (in vitro) experimental validation were excluded.

Each entry in the datasets contained the 23-nt gRNA sequence, four kinds of corresponding symbolic epigenetic features, as well as numerical and binary cleavage efficacy. The epigenetic features information was obtained from ENCODE (Consortium, 2004), including CTCF binding information obtained from ChIP-Seq assay, H3K4me3 information from ChIP-Seq assay, chromatin accessibility information from DNase-Seq assay, and DNA methylation information from RRBS assay. Each epigenetic feature was represented by an “A-N” symbolic sequence with length of 23. Here, the presence of the epigenetic feature at a particular base position of DNA regions was denoted by “A,” and its absence was represented by “N.”

Numerical cleavage efficiency of candidate gRNA was calculated using a collaborative filtering-based data normalization technique (Badaro et al., 2013). In particular, a matrix Y was formulated where each row denoted the experiments and each column represented one gRNA. *y_mn_* represented the n-th gRNA on-target cleavage efficacy in the m-th experiment. Normalized numerical gRNA on-target efficiency value was defined as

(1) $$y_{nor}=y_{mn}-(m_{row}+m_{col}+m_{all})/3$$

where *m_row_* denoted the mean value for each row, *m_col_* represented the mean value for each column, and *m_all_* denoted the mean value of Y. Next, a rank-based normalization method (Doench et al., 2016) was applied for gRNAs within each gene, and these normalized ranks were averaged across cell types, then were rescaled in [0, 1], where 1 indicated the successful on-target cleavage efficacy. The binary cleavage efficiency of gRNA was determined by using a log-fold change of 1 as the cut off, where 1 and 0 represented the high-efficiency and low-efficiency gRNAs, respectively. The processed datasets can be downloaded at https://github.com/bm2-lab/DeepCRISPR.

### Sequence Encoding

We formulated one-hot encoding to encode gRNA sequence with 23 nucleotides in length. Each base in the sequence can be encoded as one of the four one-hot vectors [1,0,0,0], [0,1,0,0], [0,0,1,0] and [0,0,0,1]. Therefore, the 1-by-23 nucleotide sequence was represented by four binary channels: A-channel, C-channel, G-channel, and T-channel. Taking A-channel as an example, the presence of the nucleotide A at a particular base pair position was denoted by 1 and the absence of the nucleotide A was represented by 0. Consequently, each gRNA was expressed by a 4 × 23 matrix, where 23 was the length of the gRNA sequence.

Analogously, epigenetic feature information including CTCF binding, H3K4me3, chromatin accessibility, and DNA methylation were represented by a 4 × 23 binary matrix. Each type of epigenetic information was denoted by a 1 × 23 matrix using “A” and “N,” with these notations meaning presence and absence of that epigenetic feature at specific position of DNA regions, respectively. To encode the epigenetic feature information, we derived a 23-length vector to encode each epigenetic feature. Thus, four epigenetic features were donated by a 4 × 23 binary matrix (see **Figure S1** for an example). The encoded sequence and epigenetic matrix of gRNA were then fed into CNN-based gRNA stream and epigenetic stream sub-networks for model training and testing.

### CNN Model Structure

We developed a CNN model to learn deep features of gRNA sequence and its corresponding epigenetic information (**Figure S2**). The proposed CNN is composed of two branches, namely gRNA stream and epigenetic stream. These two sub-networks are structurally identical, including two one-dimensional (1D) convolution layers, two average-pooling layers, and four fully connected layers.

Taking gRNA stream as an example, the input is a 4 (size of nucleotides vocabulary) × 23 (sequence length) binary matrix. The first layer of the sub-network is a 1D convolution layer (conv_1), which is designed for extracting the important local features between neighboring element values of gRNA sequence information using 256 convolution kernels of size 5. Rectified linear unit (ReLU) (Krizhevsky et al., 2012) is used as the activation function to the convolution outputs.

The second layer is a local average pooling layer (pool_1) with window size of 2 connected with the outputs of previous layer for down-sampling. Each of the average-pooling windows only outputs the average value of its respective convolution layer outputs.

The structures of the following convolution layer (conv_2) and average pooling layer (pool_2) are identical with the first (conv_1) and second (pool_1) layers mentioned above. After being flatten, the features are followed by four fully connected layers (fc_1, fc_2, fc_3 and fc_4) with the sizes of 256, 128, 64, and 40, respectively. We used dropout for model regularization to avoid overfitting.

The feature maps of the fourth fully connected layer from both gRNA and epigenetic branches are concatenated by the “concatenate” operator. Subsequently, the outputs of the concatenation layer are input to the last fully connected layer of the merged CNN network. The final output layer consists of one neuron corresponding to a regression score that highly correlates with gRNA activity. The loss function for our CNN is mean squared error (MSE) which was adapted in a previous study (Kim et al., 2018). We chose MSE because it is a good measure to prevent undesired outliers in the dataset.

### Hybrid CNN-SVR Model

We next proposed a network combining CNN and SVR called CNN-SVR to provide a data-driven and deep learning method for CRISPR/Cas9 gRNA activity prediction. For cell line-specific prediction, CNN-SVR receives a 23-nt gRNA sequence and four “A-N” symbolic epigenetic sequences with length of 23 as inputs, and it produces a regression score of gRNA on-target cleavage efficacy. Compared with machine learning-based methods that rely heavily on hand-crafted features, CNN-SVR can get rid of the dependence on manual feature engineering. The basic flowchart of CNN-SVR consists of two major stages, namely model selection and pre-training stage as well as fine-tuning and testing stage (**Figure S3**). The dataset was randomly divided into two separate sets of training and testing, respectively. One-hot encoding converts the input sequences into binary representations for downstream processing.

In the model selection and pre-training stage, there are mainly three steps: first, the encoded benchmark dataset is fed into the proposed CNN model for pre-training by the back-propagation algorithm. Randomized five-fold cross-validation tests are conducted to determine hyperparameters of the merged CNN model. Model with the minimum average validation loss is regarded as the base model. Second, the initial CNN extracted features are input to SVR classifier for training and evaluating. SVR (i.e., cost C, gamma, and epsilon) is optimized using a grid search approach to achieve the optimal performance. Third, a two-step strategy is employed to remove the redundancy of CNN features (see details in the section *Feature Representation Optimization*). The extracted low-dimensional representative feature data and their corresponding gRNA cleavage efficacy values are fed into SVR classifier for model training.

**Figure 1:**
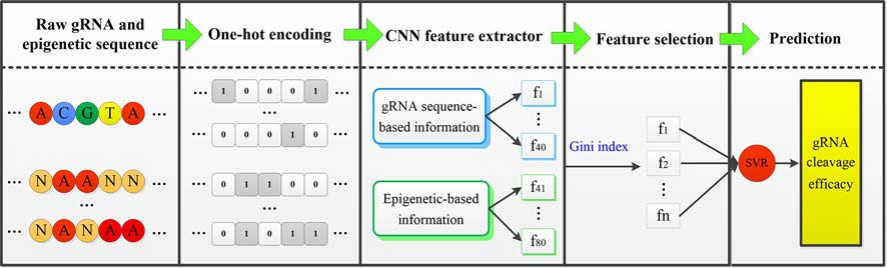
An illustration of procedures for cell line-specific gRNA on-target activity prediction based on CNN-SVR. Here, [f_1,f_2,⋯,f_n ] is the subset of [f_1,f_2,⋯,f_80 ].

In the fine-tuning and testing stage, there are mainly two steps: First, the well-trained CNN model is applied to extract features from new cell line data. Only the fourth fully connected layer of gRNA stream and epigenetic stream, and the top fully connected layer of the merged CNN are fine tuned. MSE loss function is minimized by back-propagation approach. Second, the extracted low-dimensional representative features are fed into the well-trained SVR classifier to complete the final gRNA activity prediction. **Figure 1** displayed the overall framework of our CNN-SVR; the procedures were described as follows:

- The gRNA sequence and epigenetic feature sequences are converted into two 4×23 binary matrices by one-hot encoding.
- The encoded gRNA and epigenetic sequences are fed into the well-trained CNN-based gRNA stream and epigenetic feature stream to fine-tune and extract features, respectively.
- SVR classifier is trained based on the optimal feature set. Ultimately, the well-trained SVR model assigns a prediction cleavage efficacy score for the candidate gRNA.

### Experimental Setup

To evaluate feasibility of CNN-SVR for gRNA activity prediction, we conducted numerical experiments on public datasets. We implemented our algorithms using Keras (2.1.0) with Tensorflow (1.4.0) as the backend, running on Intel Core i7 CPU at 3.6 GHz with 16 GB RAM and NVIDIA 8 GB GTX 1080 GPU. The optimized parameters were tuned automatically under the Adam optimizer (Kingma and Ba, 2014).

### Implementation of the Hybrid CNN-SVR Model

#### CNN Model Selection and Training

In the proposed architecture, the distribution of each network parameter was determined empirically. The main purpose of hyperparameter optimization was to choose a set of hyperparameters for a deep architecture, usually with the goal of optimizing performance of the architecture on an independent dataset. Grid search from the Scikit-learn Python library was adopted to tune the hyperparameters of the proposed architectures. Hyperparameter optimization experiments were performed sequentially as follows: the network weight initialization over the choice (“zero,” “he_uniform,” “uniform,” “glorot_uniform,” “lecun_uniform,” “normal,” “he_normal”), dropout regularization over the choice (0.2, 0.3, 0.4, 0.5, 0.6), batch size over the choice (64, 128, 256, 512), and number of epochs over the choice (50, 100, 200, 300).

All the constructed neural network models were trained and validated on the benchmark dataset (180512 samples). We randomly assigned the samples of the no-redundant dataset with 80% of samples for training and 20% of samples for testing with five-fold cross-validation in the training phase. Cross-validation contributed to avoiding overfitting and guaranteeing the accuracy of our model in which the datasets were divided into five equal parts randomly. In each training, one part was regarded as the testing dataset, while the remaining four parts were taken as the training dataset. Thus, we obtained 115528 training samples, 28881 validation samples, and 36103 testing samples, respectively. Separate training and validation data were applied to train the model, while the test data was used to evaluate the performance of the trained model. We chose the model that showed the minimum average validation loss as the final CNN model. After optimization, the hyperparameters were as follows: kernel_initializer: glorot_uniform; batch size: 256; epoch: 200; dropout: 0.3 (keeping 70% of the connections).

#### SVR Training and Testing

Next, CNN extracted features were fed into the SVR classifier. We implemented the SVR algorithm in Scikit-learn library. Grid search procedure was performed to find the optimal penalty parameter C, kernel parameter gamma, and epsilon. For training SVR with Gaussian radial basis kernel (RBF) classifier, grid search range of each parameter was as follows: cost C from the choice (1.0,1.1,⋯,1.9), kernel coefficient gamma over the choice (0.11,0.12,⋯,0.15), epsilon from the choice (0.08,0.09,⋯,0.12). We selected the parameters that maximized the maximum average area under ROC curve (AUROC) value as the final parameters of SVR classifier. The optimized parameters of the SVR were as follows: C was 1.7, gamma was 0.12, epsilon was 0.11. These parameters were then used to train the CNN-SVR model.

#### Feature Representation Optimization

Considering that CNN extracted features might introduce redundancy which can undermine model performance, we employed a two-step feature optimization strategy to identify important feature subsets from the initial CNN features. To be specific, we first applied random forest to the learnt feature representation from well-trained CNN model and obtained the ranked feature list based on information gain (Liaw and Wiener, 2002). We trained the random forest model with 250 decision trees using Scikit-learn. The feature importance distribution of the top 20 features based on their importance scores was illustrated in **Figure S4**. As can be seen, the seventeenth feature of CNN extracted initial features was the most predictive feature. Second, the sequential forward search (SFS) (Whitney, 2006) was performed to determine the optimal feature set. We gradually added features from random forest feature rank from higher score (lower rank) to lower score (higher rank) to reconstruct the SVR models. The feature subset with the relatively higher value of AUROC was regarded as the optimal feature set. We used the AUROC since it is a good indicator to evaluate the real performance of models. We noted that, when the feature number reached at 13, the model achieved the maximum AUROC of 0.9769. Hence, the top 13 features (i.e., “feat_17,” “feat_26,” “feat_9,” “feat_19,” “feat_30,” “feat_6,” “feat_12,” “feat_39,” “feat_36,” “feat_21,” “feat_22,” “feat_3,” “feat_25”) in the random forest rank list were integrated into SVR classifier to train the prediction scheme. Here, “feat_17” means the 17th feature of CNN extracted initial features (total 80 features). Thereby, we carried out the determined hyperparameters by integrating the optimal features on the benchmark dataset under five-fold cross-validation to obtain the well-trained CNN-SVR model. The training data, validation data, and testing data were built consistent with the above mentioned data partitioning way in the *CNN Model Selection and Training* section. The well-trained CNN-SVR reached an overall Spearman correlation of 0.952, AUROC value of 0.977.

#### Transfer Learning for New Cell Line Specific Prediction

In this section, we proposed a fine-tune strategy by borrowing information from the benchmark data, aiming at boosting the prediction performance on small sample size cell line-specific data. To this end, four above cell-line datasets were combined together for model training and testing. We constructed the training, validation, and test data from total four datasets based on gRNA sequence composition and epigenetic feature information. The training data (13401 samples) and test data (3748 samples) were also generated in the same way in the *CNN Model Selection and Training* section. Randomized five-fold cross-validation was implemented in the training phase.

Considering training a full CNN network with small number of cell line data may result in overfitting, which may lead to poor performance. Transfer learning (Bengio, 2012) is effective to address the challenge where the learned parameters of well-trained networks on a large dataset are shared. The main idea of transfer learning is to use a pre-trained model which is trained on large dataset and to transfer its well-trained parameters (e.g. weights) to the targeted network model. Though the dataset is different from the one that the network was trained on, the lower-level features are similar. Thus, the last fully connected layers are usually trained on the new dataset. Transfer learning has been widely applied to computer vision (Shin et al., 2016; Cheng and Malhi, 2017) and achieved a valuable efficacy in terms of accuracy. We applied transfer learning from the benchmark dataset pre-trained CNN model, and fine-tuned for small sample cell line data. Note that, the low-level features between the benchmark data and cell line-specific data are similar. Therefore, we froze the convolution layers, average pooling layers and the first three fully connected layers of both gRNA stream and epigenetic stream. After borrowing weights of the well pre-trained CNN base network, we fine-tuned the weights of the last fully connected layers of both gRNA and epigenetic sub-networks and those of the merged fully connected layer to optimize the mean validation squared error loss function. During fine tuning, we only updated 5281 free parameters. By fixing the weights parameters in the other layers, CNN-SVR could prevent overfitting and effectively learn to integrate the sequence representative and epigenetic information. For any given cell line of interest, the training process was described as follows:

- Pre-train a CNN model with the benchmark data for 200 epochs.
- Freeze the convolution layers, average pooling layers, the first three fully connected layers (for both the gRNA stream and epigenetic stream).
- Train the fourth fully connected layer of the above two streams and the top fully connected layer of the merged CNN model with training data from the cell line of interest for another 200 epochs.
- Evaluate the model on the test data.

### Settings of Other Methods

For the L1-regularized linear regression (L1), we applied LassoCV from Scikit-learn Python library to find out the optimal parameters of alpha by cross-validation. Grid searching range of regularization parameter alpha was (0.01, 0.02,⋯,0.1). Other parameters were set with default values. We achieved an optimal value of 0.01. Similarly, we applied RidgeCV and ElasticNetCV with the same grid searching range of L1 to set parameter alpha for L2-regularized linear regression (L2) and L1L2-regularized linear regression (L1L2), respectively. After optimization, the best alpha values of L2 and L1L2 were 0.04 and 0.01, respectively. These parameters were then used to train the CNN-L1, CNN-L2, and CNN-L1L2 models. Other parameters of L2 and L1L2 were set with default values.

We ran the code of Seq_deepCpf1 using the same data and basic training process (downloaded from GitHub at https://github.com/MyungjaeSong/Paired-Library). Note that, the input of Seq_deepCpf1 was a 4-by-34 dimensional binary matrix. Here, we changed the input shape of Seq_deepCpf1 model into 4-by-23 to match the size of the data in this study. Besides, we used the benchmark dataset to pre-train the Seq_deepCpf1 model. To make a fair comparison, we only fine-tuned the weights parameters in the last two layers (1681 free parameters) for cell line-specific prediction. The numerical experimental condition was set consistent with DeepCRISPR. The source codes of DeepCRISPR were downloaded from https://github.com/bm2-lab/DeepCRISPR. SSC, sgRNA Designer and WU-CRISPR provided available web based applications. More details can be found in **Table S1**.

### Performance Measurements

To quantitatively evaluate the performance of our CNN-SVR, Spearman correlation coefficient between predicted and measured on-target activity was calculated. We chose Spearman correlation is due to it is more robust to outliers than Pearson’s correlation coefficient (Mukaka, 2012). Besides, it was adapted in previous studies (Doench et al., 2016; Chuai et al., 2018; Kim et al., 2018). Spearman correlation was calculated using SciPy library (http://scipy.org). In addition, AUROC was employed to comprehensively quantify the overall predictive model performance. The value of AUROC ranges from 0.5 to 1. A larger AUROC value represents that model achieves better and more robust performance. Note that, we used 0.5 AUROC as the baseline. Statistical test was performed using SciPy library for comparing the differences between GC content distributions of different datasets. Two-sample Kolmogorov–Smirnov test was used for testing the distance between two distributions under the null hypothesis that samples from the same continuous distribution. P < 0.05 was considered to indicate statistically significant difference.

## Results

### Comparison CNN-SVR With CNN Model

To verify the feasibility of our approach, we compared our CNN-SVR with CNN model on the above four cell-line datasets. The current practice of training a model was to use cell-line specific data for prediction. Each data set was randomly split into a training set and an independent testing set with 80% and 20% classes. **Table 1** summarized the results regarding evaluation criteria including Spearman correlation and AUROC under 10-round 10-fold cross-validation tests. CNN-SVR showed substantially better performance in terms of Spearman correlation. As for AUROC, CNN-SVR was superior to CNN on datasets HEK293T, HELA, and HL60. These results showed that CNN-SVR is more predictive than CNN for gRNA on-target activity, further conforming the feasibility and effectiveness of the combination of CNN and SVR classifier.

**Table 1:** Performance comparison between CNN-SVR and CNN models for gRNA activity prediction on four cell-line datasets under 10-time 10-fold cross-validation.

Model	CNN-SVR	CNN	CNN-SVR	CNN
	Spearman		AUROC	
HCT116	**0.719 ± 0.008**	0.661 ± 0.030	**0.933 ± 0.001**	0.932 ± 0.001
HEK293T	**0.807 ± 0.016**	0.725 ± 0.029	**0.983 ± 0.002**	0.972 ± 0.001
HELA	0.699 ± 0.006	**0.702 ± 0.007**	**0.933 ± 0.001**	0.916 ± 0.001
HL60	**0.589 ± 0.006**	0.576 ± 0.040	**0.934 ± 0.003**	0.914 ± 0.003

Performance is shown as mean ± standard deviation. This representation also applies to **Table 2**. The best performance across different folds cross-validation method is highlighted in bold for clarification. These highlights also apply to **Tables 2** to **4** and **Tables S3** to **S5**.

### Comparison of Various CNN Combined Regression Models

We then attempted to access the regression performance of CNN-SVR. To this end, we compared CNN-SVR with three CNNs plus regression approaches, including CNN plus L1 (CNN-L1), CNN plus L2 (CNN-L2), and CNN plus L1L2 (CNN-L1L2) on the above four cell lines datasets. Note that for each cell line, the training data and test data were generated in the same way as described in the section *Comparison CNN-SVR With CNN Model*. Ten-time 10-fold cross-validation tests were randomly performed and the average of the individual performance were summarized in **Table 2**. Overall, CNN-SVR performed better than CNNs with different regression methods on all datasets. These observations revealed that the regression learning performance of our SVR surpasses other regression methods on gRNA activity prediction.

**Table 2:** Performance comparison of CNN-SVR and different CNNs combined regression models for gRNA activity prediction on four cell-line datasets under 10-time 10-fold cross-validation.

Model	HCT116	HEK293T	HELA	HL60
**(A) Spearman correlation**				
CNN-SVR	**0.719 ± 0.008**	**0.807 ± 0.016**	**0.699± 0.006**	**0.589± 0.006**
CNN-L1	0.712± 0.010	0.793 ± 0.004	0.633± 0.020	0.542± 0.033
CNN-L2	0.670± 0.025	0.731 ± 0.032	0.683± 0.009	0.517± 0.034
CNN-L1L2	0.701± 0.008	0.803 ± 0.012	0.682± 0.005	0.589± 0.018
**(B) AUROC**				
CNN-SVR	**0.933 ± 0.001**	**0.983 ± 0.002**	**0.933 ± 0.001**	**0.934 ± 0.003**
CNN-L1	0.931 ± 0.001	0.982 ± 0.001	0.924 ± 0.002	0.930 ± 0.003
CNN-L2	0.919± 0.002	0.975 ± 0.002	0.923± 0.002	0.895± 0.008
CNN-L2	0.918± 0.003	0.977 ± 0.001	0.915± 0.002	0.912± 0.004

The tables from top to bottom respectively record the Spearman correlation and AUROC of CNN-SVR and three CNN combined regression methods.

### Comparison With State-Of-the-Art Methods

To validate the performance of proposed CNN-SVR, we compared it with one deep learning-based method (DeepCRISPR) and three machine learning methods including sgRNA Designer, SSC, and WU-CRISPR (**Table S2**). Note that Seq-deepCpf1 only allows for receiving gRNA sequence as input. So, this approach was not compatible with other methods when considering both gRNA sequence and epigenetic information. To make a fair comparison, we trained CNN-SVR model based on the training data strictly consistent with other methods. The above four datasets were used for performance evaluation. For each cell line, the training and test data were constructed in the same way as described in the section *Comparison CNN-SVR With CNN Model*. For any given cell line of interest, the training data was built by integrating all the training data from four cell lines. The performance was evaluated on each cell line-specific testing set, respectively.

**Figure 2:**
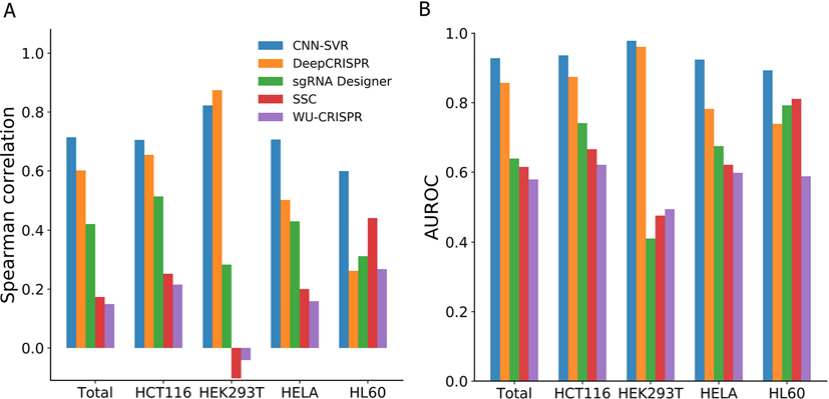
Performance comparison of CNN-SVR and other prediction models on various testing cell line data.

On the whole, CNN-SVR achieved the highest average Spearman correlation (**Figure 2A**). Specifically, CNN-SVR exhibited better Spearman correlation on three datasets (i.e., Total, HCT116, HELA and HL60), whereas for dataset HEK293T, it performed slightly worse than DeepCRISPR. **Figure 2B** illustrated the performance in terms of AUROC. Some interesting conclusions can be extracted from this figure. First, deep learning models were superior to machine learning methods. Second, CNN-SVR exhibited better predictive power than another deep learning model DeepCRISPR. The details of their performance can be found in **Table S3**. To sum up, these observations indicated that CNN-SVR outperforms the compared state-of-the-art methods for predicting gRNA on-target activity.

### Assessment of Generalization Performance With a Leave-One-Cell-Out Procedure

Next, we investigated the generalizability ability of CNN-SVR in new cell types. For this purpose, we took turns to test the model on the above four cell-line datasets using a leave-one-cell-out approach. The training data and test data for each cell line were built in advance. Note that, the partitioning method for each cell line data followed the way illustrated in the section *Comparison CNN-SVR With CNN Model* In the training phase, for a given cell line to be predicted, we just used the training data from all other three cell lines (lacking training data of given cell-line of interest). In the testing stage, we evaluated the performance on the test data of the given cell-line of interest. Taking leave-HCT116-out procedure as an example, we trained the model by combining training data of HEK293T, HELA and HL60 cell lines (without HCT116 cell line training data), and evaluated the model on HCT116 cell line testing set. For fair comparison, we tested the proposed CNN-SVR under the same condition with DeepCRISPR, sgRNA Designer, SSC, and WU-CRISPR on the four cell-line datasets.

**Figure 3:**
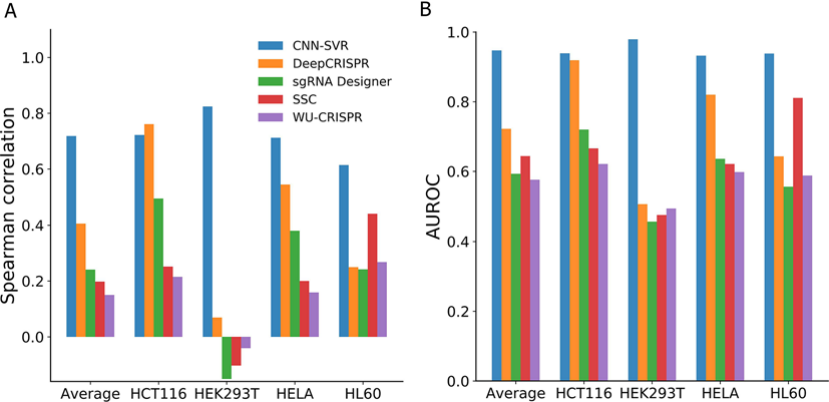
Performance comparison of CNN-SVR and other prediction models on various testing cell line data with a leave-one-cell-out procedure.

As can be seen from **Figure 3A**, among the compared models, CNN-SVR exhibited the best predictive power, with average Spearman correlation of 0.714. Compared with DeepCRISPR, which was one of the best state-of-the-art approaches, CNN-SVR showed superior performance on all datasets except for dataset HCT116. DeepCRISPR got comparable performance with CNN-SVR on HCT116 dataset. Furthermore, CNN-SVR outperformed other methods on all datasets in terms of AUROC (**Figure 3B**). Together, these results demonstrated the excellent generalizability of CNN-SVR. More details of the performance can be found in **Table S4**.

### Evaluation of Robustness of Prediction Models

In this section, we aimed to compare the robustness of the above methods. To this end, we examined the changes between Spearman correlation and AUROC values obtained by training with four cell datasets (**Figure 2**) and those produced by the leave-one-cell-out approach (**Figure 3**). For each evaluation criterion, we calculated the difference of each model by subtracting the results of training with leave-one-cell-out (**Table S4**) from the cell-line independent (**Table S3**). Taking CNN-SVR as an example, the AUROC difference of HCT116 dataset was calculated as follows:

(2) $$\Delta {\mathrm{AUROC}}_{\mathrm{CNN}-\mathrm{SVR}}=0.936-0.939=-0.003$$

where “ΔAUROC” means the difference value of AUROC. It can be seen that our CNN-SVR substantially showed smaller changes than DeepCRISPR in terms of the above mentioned two evaluation measures (**Table 3**). Interestingly, we observed that the performance of DeepCRISPR on dataset HEK293T using the whole training set was significantly better than that by leave-one-cell-out approach (with Spearman correlation difference value of 0.805, AUROC difference value of 0.455). Previous studies have shown that gRNAs with low or high GC content tended to be less active (Doench et al., 2014; Wang et al., 2014). We analyzed GC content of the four cell datasets. As expected, dataset HEK293T has the lowest GC content (vs. dataset HCT116, P=1.35E-52; vs. dataset HELA, P=1.14E-69, vs. dataset HL60, P=1.45E-07, two-sample Kolmogorov-Smirnov test, **Figure S5**).

**Table 3:** The differences of Spearman correlation and AUROC between independent test and a leave-one-cell-out approach between CNN-SVR and DeepCRISPR.

Model	HCT116	HEK293T	HELA	HL60
**(A) Spearman correlation**				
CNN-SVR	**-0.017**	**-0.002**	**0.011**	-0.015
DeepCRISPR	-0.107	0.805	-0.043	**0.012**
**(B) AUROC**				
CNN-SVR	**-0.003**	**-0.001**	**-0.008**	**-0.045**
DeepCRISPR	-0.045	0.455	-0.038	0.096

### Effect of Epigenetic Features on gRNA Cleavage Efficacy

In this section, we determined whether cell line-specific epigenetic features really boost the predictive performance. We examined the performance of deep learning-based methods on the four cell-line datasets only considering gRNA sequence composition and compared them with those considering both gRNA sequence and epigenetic information (see the section *Assessment of Generalization Performance With a Leave-One-Cell-Out Procedure*). We trained the prediction models without epigenetic information (sequence only) for each cell line with a leave-one-cell-out procedure. Note that, we trained the model just considering the gRNA stream. Other numerical experimental conditions were in accord with the section *Assessment of Generalization Performance With a Leave-One-Cell-Out Procedure*. For fair comparison, we compared our methods with two deep learning-based methods (i.e., DeepCRISPR and Seq-deepCpf1) only considering sequence composition.

**Figure 4:**
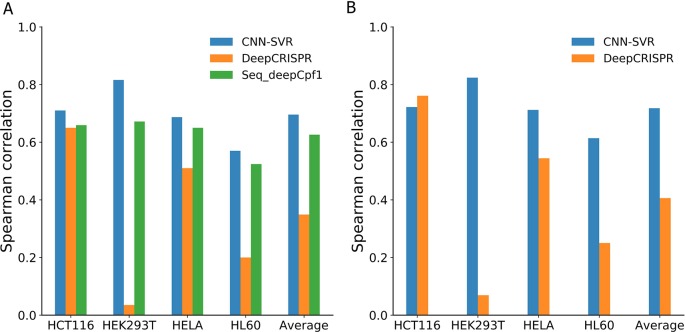
Spearman correlation between different deep learning-based models and datasets. Models considering **(A)** gRNA sequence composition only and **(B)** both gRNA sequence and epigenetic information.

**Figure 4** and **Table 4** compared the prediction performance of various deep learning methods trained using different datasets. Two interesting conclusions can be drawn as below. First, CNN-SVR showed better performance compared with other models. Second, as expected, the prediction accuracies of models trained only considering sequence composition (**Figure 4A** and **Table 4A**) became lower compared with those trained with both sequence and epigenetic data (**Figure 4B** and **Table 4B**). To conclude, these observations confirm that cell line-specific epigenetic features contribute to gRNA activity and specificity. More details of their performance of Spearman correlation can be found in **Table S5**.

**Table 4:** AUROC of different deep learning-based methods by considering gRNA sequence only and incorporating both gRNA sequence and epigenetic features.

Model	HCT116	HEK293T	HELA	HL60	Average
**(A) Sequence-only**					
CNN-SVR	**0.938**	**0.976**	**0.930**	**0.928**	**0.943**
DeepCRISPR	0.887	0.474	0.788	0.584	0.683
Seq-deepCpf1	0.931	**0.976**	0.925	0.920	0.938
**(B) Sequence composition and epigenetic features**					
CNN-SVR	**0.939**	**0.979**	**0.932**	**0.938**	**0.947**
DeepCRISPR	0.919	0.506	0.820	0.643	0.722

### Visualizing Importance of Position-Specific Nucleotides

**Figure 5:**
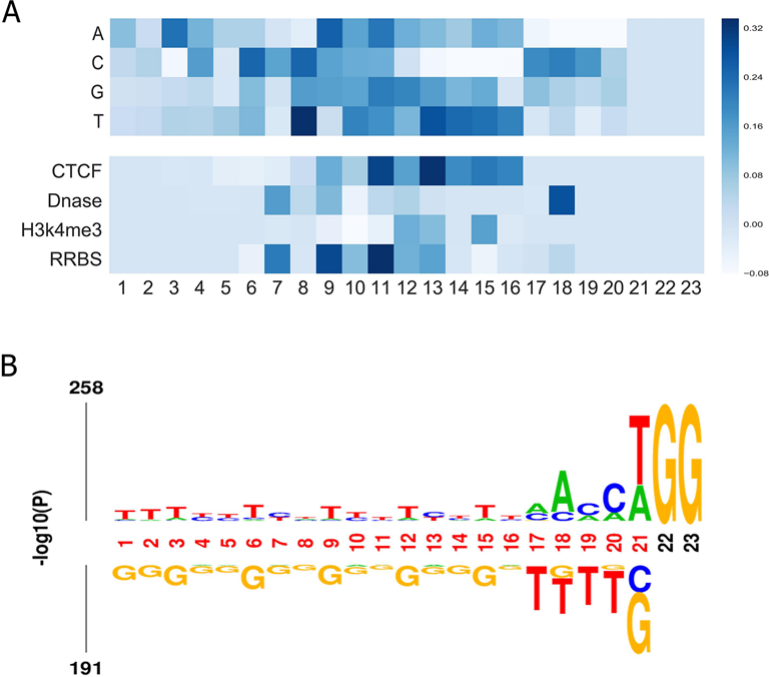
**(A)** Visualization of the importance of different nucleotides and epigenetic features at different positions for our model trained on the benchmark dataset. The colors represent the contribution of the position-specific nucleotides to determining an efficient gRNA. The x-axis shows the positions of the nucleotide in the sequence. The y-axis lists all possible nucleotides. This representation also applies to. **(B)** Preference of nucleotide sequences that impact CRISPR/Cas9 gRNAs activity.

Finally, we aimed to investigate what sequence patterns of gRNA contribute to its on-target activity. Using the method in a previous study (Xie et al., 2013), we investigated the feature importance of all possible position-specific nucleotides. In brief, we constructed a specific sequence and its corresponding epigenetic features to feed the well-trained CNN model and took the outputs for visualization. More details can be found in **Supplementary Material**. **Figure 5A** depicts the importance of all four nucleotides and epigenetic features at different positions. Several interesting results can be observed: (i) Most of the top features were generated by convolving the middle region of input matrix. (ii) Thymines are found to be disfavored at the fourth position adjacent to the PAM. The same observation was obtained by Chuai et al., (2018), which is consistent with previous finding that multiple uracils in the spacer result in low gRNA expression (Doench et al., 2014). Another study also found that thymine in the seed sequence might destabilize interactions between the protein and crRNA (Kim et al., 2017). (iii) Cytosine is informative at 3-nt upstream of the PAM since the cleavage site usually resides 3 nt upstream the PAM. (iv) Our model suggests that cytosine is also preferred at position 17, which coincides with a previous finding that the cleavage is 3 nt, 4 nt or even further upstream of the PAM (Shou et al., 2018). (v) In general, the middle region contains more information of the epigenetic features. Notably, 3 nt upstream of the PAM has a consistent preference for opening-chromatin information of Dnase. This observation is in accordance with a previous study, which corroborates that consideration of target site accessibility can boost the accuracy of gRNA activity prediction (Kim et al., 2018). Besides, we presented the sequence logo to visualize the nucleotide differences on the benchmark dataset. Overall, the result is in line with our feature analysis (see **Figure 5B**).

We also explored the importance of dimers. Here, by adopting the method proposed above, we generated a sequence which only contains one dimer (out of 16 possible dimers) at every position k and repeated the aforementioned process for all subsequences. The scores of all the constructed subsequences for all the positions were plotted as a heatmap in **Figure S6**. We note that most of the top features were generated by convolving the region of the seed sequence of the gRNAs. This observation coincides with previous finding that a prototypical 10–12 nt PAM-proximal seed sequence largely determines target efficacy (Jinek et al., 2012; Cong et al., 2013).

## Discussion

Accurate prediction of gRNA cleavage efficacy is pivotal to understanding the mechanisms of CRISPR/Cas9 system. Although computational prediction of gRNA cleavage efficiency has made much progress recently, the accuracy remains to be improved. In this study, we introduced a novel and interpretable deep learning framework named CNN-SVR for CRISPR/Cas9 gRNA on-target activity prediction. Specifically, CNN works as a trainable feature extractor and SVR performs as a gRNA cleavage efficacy predictor. Compared with CNN and three CNNs combined regression-based algorithms, CNN-SVR achieved the best performance. CNN-SVR could not only automatically extract gRNA sequence and the corresponding epigenetic features using the CNN, but also improve the generalization ability of CNN and regression accuracy.

Previous studies suggested that ensemble learning (Woźniak et al., 2014) by incorporating multiple neural networks together can achieve higher accuracy than a single learner (Maqsood et al., 2004). Inspired by this, instead of using a single convolution network to train the feature vectors of gRNA like Seq-deepCpf1, we merged two sub-networks (i.e., gRNA stream and epigenetic stream) to train gRNA sequence and its corresponding epigenetic information. In addition, the architecture of the proposed sub-networks was considerably shallower than DeepCRISPR. Compared with several current state-of-the-art learning-based methods, CNN-SVR can effectively exploit deep features of gRNA sequences. Experimental results demonstrated the power of our CNN-SVR for CRISPR/Cas9 gRNA activity prediction.

Besides, we found our CNN-SVR system has good generalizability in new cell types by using leave-one-cell-out approach on the four testing datasets. By analyzing the changes of prediction results with and without considering epigenetic information, we observed that considering of epigenetic features slightly improves the accuracy of CRISPR/Cas9 gRNA activity prediction. This result was consistent with previous studies (Chen et al., 2017; Chuai et al., 2018; Kim et al., 2018). Chuai et al. found that the prediction on HEK293T became poor (Chuai et al., 2018). They speculated that it was mainly due to the insufficient training data of the HEK293T training dataset. Note that our findings suggested that the reducing epigenetic features may be one possible explanation for the observation. Additionally, the low GC content of dataset HEK293T may be another possible explanation. We concluded that our CNN-SVR gained better generalization and robustness than DeepCRISPR.

Our model focused on gRNA sequence and four kinds of epigenetic features for CRISPR/Cas9 on-target prediction. A recent study on protein-related prediction has shown that integration of other manual extracted features, such as molecular weight and hydrophobicity into the deep learning model could improve the predictive power (Wang et al., 2016). It has been reported that GC content is associated with gRNA activity (Doench et al., 2014; Wang et al., 2014). We thus made a preliminary exploration of adding this sequence-derived feature with our CNN-SVR for gRNA activity prediction on the above four datasets. Note that, the training data and test data were constructed in the same way as described in the section *Comparison CNN-SVR With CNN Model*. Overall, addition of GC content to CNN-SVR increased the predictive ability, with Spearman correlation coefficients of 0.645, 0.656 and 0.608 on datasets HCT116, HELA and HELA, respectively. Detailed results can be found in **Table S6**. Therefore, manual design of proper gRNA features will contribute to the prediction ability. In the future, we plan to develop deep learning models incorporating indirect sequence-derived sequence features to improve the prediction performance, such as chromatin accessibility (Kim et al., 2018), RNA thermodynamics (Abadi et al., 2017), secondary structure of gRNA (Abadi et al., 2017), and GC content, which cannot be automatically obtained by deep learning models.

Visualization method was applied to our model. Note that the PAM and the core region (1-5 nt adjacent to the PAM) are very important for gRNA target efficacy. However, we observed that the most top features were generated by convolving the middle region of the input matrix. Therefore, we believe expanding the upstream and downstream of the target sequence in a proper length can enhance the generalization performance of the model. For example, Kim et al. found 34 bp (4 bp + PAM + 23bp protospacer + 3bp) was adequate as the input sequence of their models in CRISPR/Cpf1 system (Kim et al., 2018).

Several future improvements are expected. First, in the present study, taking advantage of CNN and SVR, we designed the relative concise hybrid CNN-SVR architecture. Research on the deep learning-based model for CRISPR/Cas9 system gRNA cleavage efficiency prediction is still at an early stage. Numerous complex and modern deep learning models await exploration. Second, as pre-training technique has great influence on the final predictive performance, therefore critical to know on what a model was trained before use. In general, sequencing-based models are more general applicable, but are only capable of predicting the genotype changes rather than functional result. On the contrary, phenotypic trained models are fit for recognizing target sites that cause functional changes but limited to numerical experiments with the same condition as the training set. However, the amount of available gRNA knockout data is relatively small, which provides a big challenge for training the deep learning model. Consequently, appropriate data augmentation techniques are needed to increase the training sample size. Third, reasonable encoding schemes, which provide maximum biological characteristics information as well as reducing the compute costs, will boost the CRISPR/Cas9 gRNA activity prediction accuracy. Finally, it is possible that integration of manual extracted features associated with gRNA activity can also improve predictive power of deep learning models.

## Conclusions

In this study, we present CNN-SVR, an efficient and extendable method to automatically learn the sequence features for CRISPR/Cas9 gRNA activity prediction. We adopt a merged CNN architecture for gRNA and its corresponding epigenetic features extraction, and subsequently incorporate SVR classifier to predict gRNA cleavage efficiency. Compared with CNN, two state-of-the-art deep neural network based models (e.g. DeepCRISPR and Seq-deepCpf1) and three machine learning tools (i.e., sgRNA Designer, SSC, and WU-CRISPR), CNN-SVR can effectively exploit features interactions from feed-forward directions to learn deeper features of gRNAs and their corresponding epigenetic features. Experimental results on the published datasets demonstrate the superiority of our CNN-SVR for CRISPR/Cas9 gRNAs on-target activity prediction.

## Data Availability Statement

Publicly available datasets were analyzed in this study. This data can be found here: https://github.com/Peppags/CNN-SVR.

## Author Contributions

All authors contributed to the project design. GZ wrote the analysis source code, analyzed the data, and drafted the full manuscript. ZD and XD critically revised the final manuscript. All authors read and approved the final manuscript.

## Funding

This research was funded by the National Natural Science Foundation of China (NSFC) (Grant 61872396, 61872395 and U1611265), and also by Pearl River Nova Program of Guangzhou (201710010044).

## Conflict of Interest

The authors declare that the research was conducted in the absence of any commercial or financial relationships that could be construed as a potential conflict of interest.
