# Supplementary material for: A Novel Hybrid CNN-SVR for CRISPR/Cas9 Guide RNA Activity Prediction
Source: Front Genet. 2020 Jan 8;10:1303. doi: 10.3389/fgene.2019.01303 (PMC6960259; doi:10.3389/fgene.2019.01303)
Supplement: Supplementary file 1 [file DataSheet_1.docx]

Supplementary Material

# Supplementary Figures and Tables

## Supplementary Figures


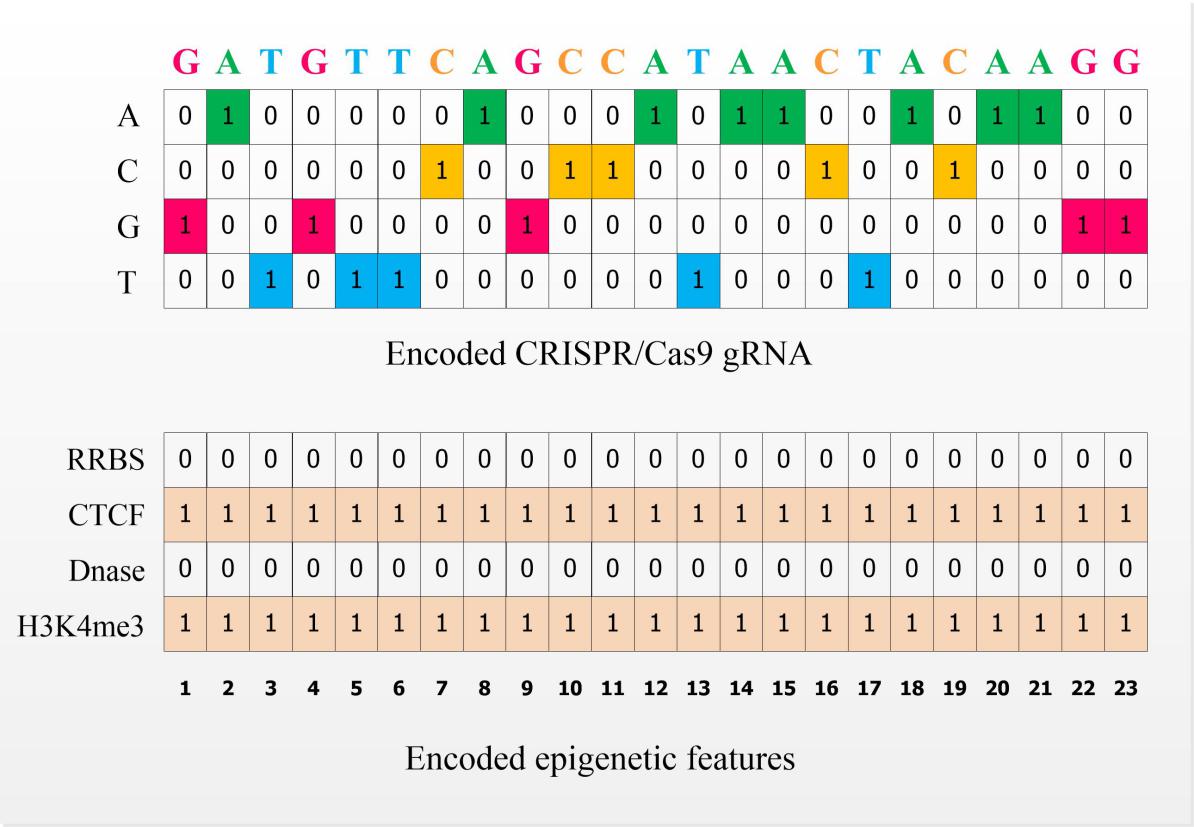


**Supplementary Figure 1.** An example of the one-hot encoding representation schema for gRNA and its corresponding epigenetic features. The 1-by-23 CRISPR/Cas9 gRNA sequence is denoted by four binary channels: A-channel, C-channel, G-channel, and T-channel. For specific nucleotide channel, its presence at one particular position is denoted by 1, while the absence is represented by 0. Similarly, four corresponding 1-by-23 epigenetic sequences including CTCF, H3K4me3, Dnase and RRBS are collectively represented by a $4\times23$ binary matrix. The presence and absence of that epigenetic feature at specific position of DNA regions are represented by 1 and 0, respectively.


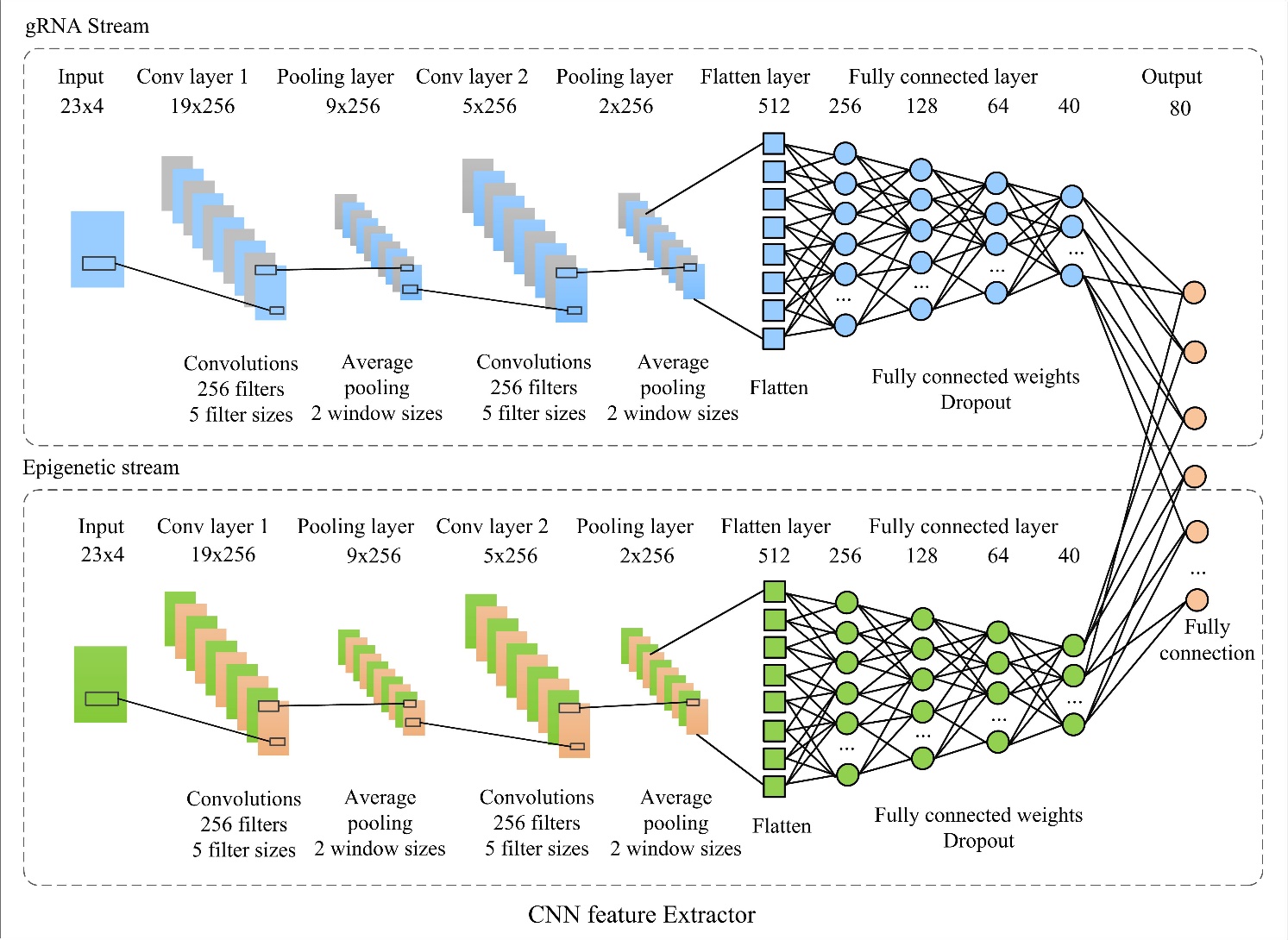


**Supplementary Figure 2.** The architecture of the CNN features extractor. The network contains a gRNA sub-network for extracting features from gRNA and an epigenetic stream sub-work for extracting features from four epigenetic features. Note that, both of these two sub-networks are structurally identical, including two one dimensional (1D) convolution layers, two average-pooling layers and four fully connected layers. Taking gRNA-net as an example, it accepts the 4 (size of nucleotides vocabulary) × 23 (sequence length) binary matrix as input. The first layer is a 1D convolution layer (conv_1), which is applied to extract the gRNA features using 256 convolution kernels of size 5. Rectified linear unit (ReLU) is subsequently used as the activation function to the convolution outputs. The average-pooling layer (pool_1) applying a filter with window size 2 to the previous layers, are used to reduce the number of parameters. The structures of the following convolution layer (conv_2) and average pooling layer (pool_2) are consistent with the conv_1 layer and pool_1 layer, respectively. Outputs of the pool_2 layer are joined together into one vector by flattening. After that, the features are followed by four fully connected layers (fc_1, fc_2, fc_3 and fc_4) with the sizes of 256, 128, 64 and 40, respectively. The feature maps of the fourth fully connected layer from both gRNA and epigenetic branches are concatenated by “concatenate” operator. The outputs of the concatenation layer are input to the top fully connected layer of the merged CNN network. The final output layer consists of one neurons corresponding to a regression score that highly correlates with gRNA activity. Dropout is applied for the model regularization. The dropout rate will be determined in Section 2.6.1.


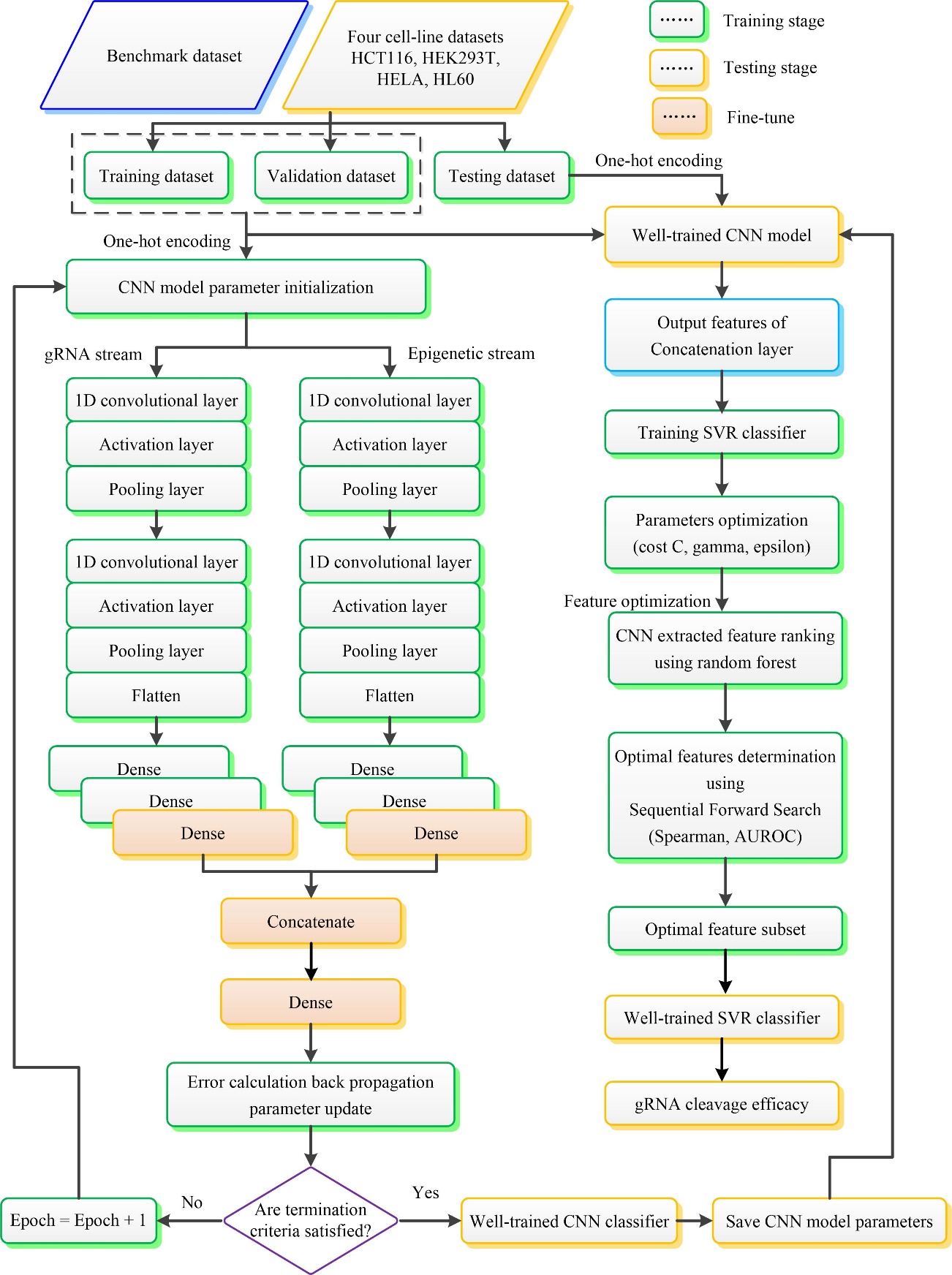


**Supplementary Figure 3.** Diagram of our proposed CNN-SVR structure for CRISPR/Cas9 gRNA on-target activity prediction.


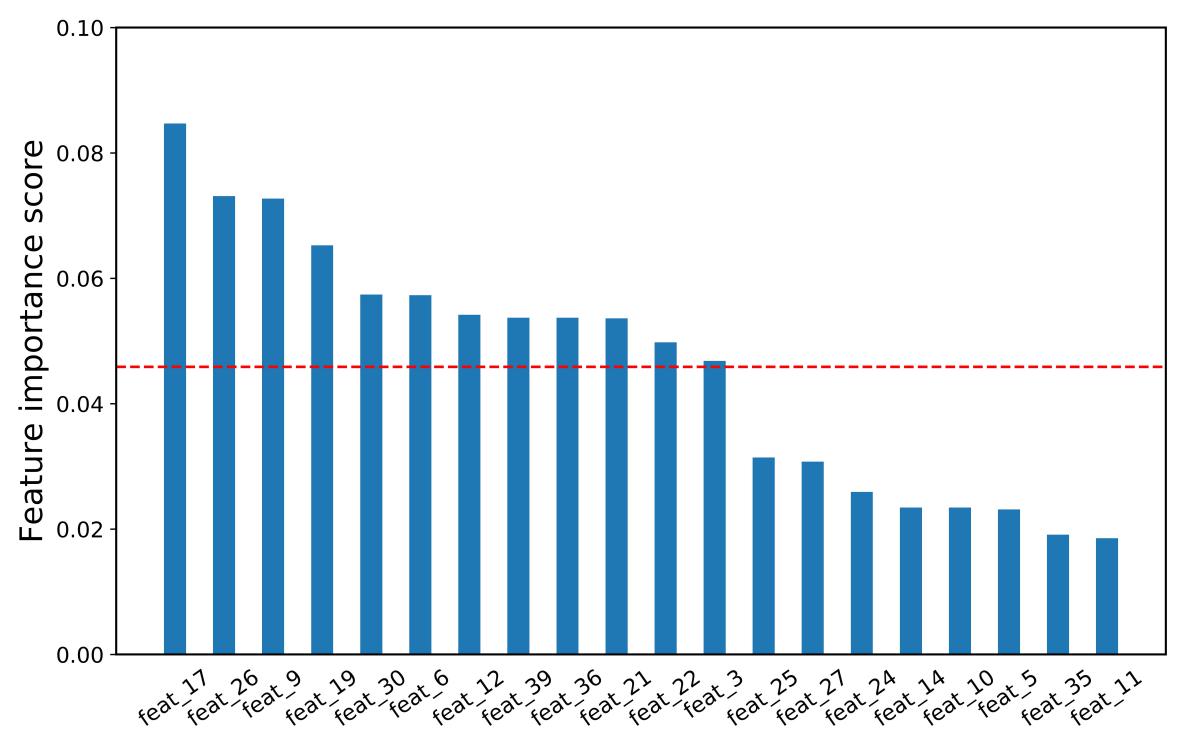


**Supplementary Figure 4.** Top 20 features from random forest importance ranking. X-axis denotes the importance scores. Y-axis represents the analyzed features. Here, ‘feat_17’ is referred to the seventeenth feature extracted by well-trained CNN model. The red dotted line shows the average importance scores of the top 20 features.


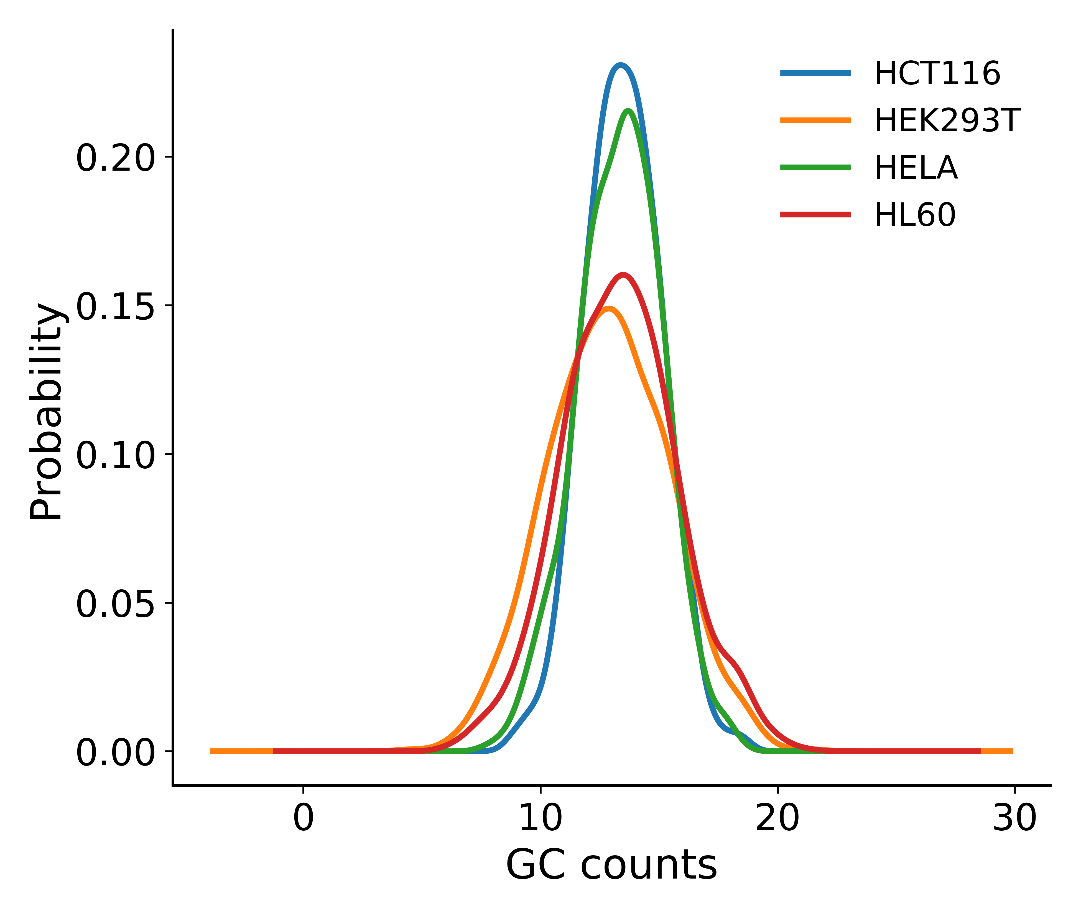


**Supplementary Figure 5.** The frequency distribution of GC content of datasets HCT116, HEK293T, HELA and HL60. Distributions are given as kernel density estimates of GC counts for each cell line-specific data. The frequency distribution of dataset HEK293T is skewed towards low GC counts end, which suggests that low GC counts are more likely to have less on-target efficiency.


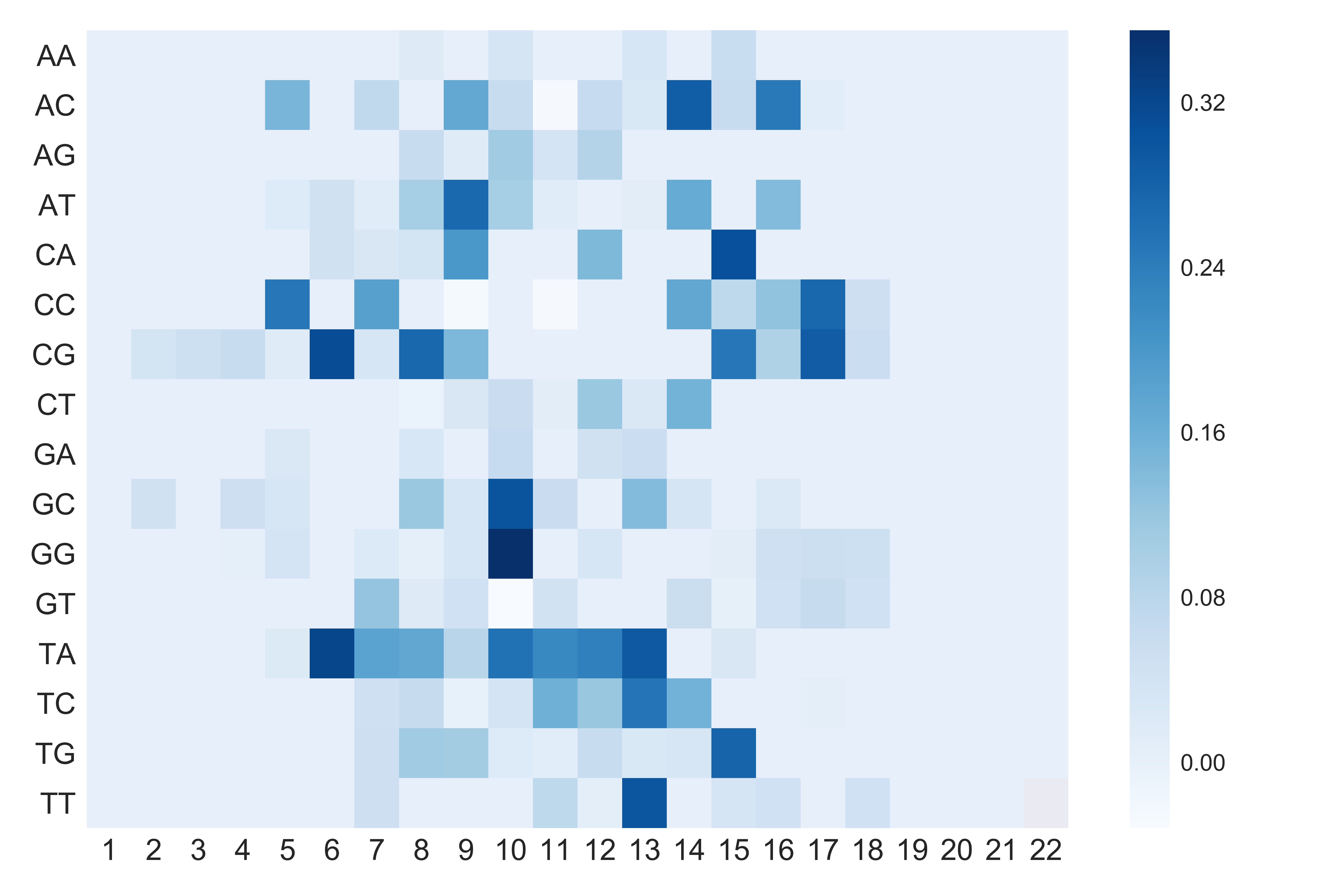


**Supplementary Figure 6.** Visualization of the importance of 16 dimers at different positions for our well-trained CNN model. The colors represent the contribution of the position-specific dinucleotides to determining an efficient gRNA. The darker blue, the more important the dimers at that position. The x-axis shows the positions of the nucleotide in the sequence. The y-axis list all possible dinucleotides.

## Supplementary Tables

**Supplementary Table 1.** Details of the gRNA activity design tools in our comparison study.

| **Tool** | **GitHub** | **Website** | **Reference** |
| --- | --- | --- | --- |
| Seq_deepCpf1 | https://github.com/MyungjaeSong/Paired-Library | http://deepcrispr.info | (Kim et al., 2018) |
| DeepCRISPR | https://github.com/bm2-lab/DeepCRISPR | http://www.deepcrispr.net/ | (Chuai et al., 2018) |
| sgRNA Designer | https://www.nature.com/articles/nbt.3437#supplementary-information | http://www.broadinstitute.org/rnai/public/analysis-tools/sgrna-design | (Doench et al., 2016) |
| SSC | http://sourceforge.net/projects/spacerscoringcrispr/ | http://crispr.dfci.harvard.edu/SSC | (Xu et al., 2015) |
| WU-CRISPR | https://github.com/wang-lab/sgDesigner | http://crispr.wustl.edu | (Wong et al., 2015) |

**References**

Chuai, G., Ma, H., Yan, J., Chen, M., Hong, N., Xue, D., et al. (2018). DeepCRISPR: optimized CRISPR guide RNA design by deep learning. *Genome Biol* 19(1)**,** 80. doi: 10.1186/s13059-018-1459-4.

Doench, J.G., Fusi, N., Sullender, M., Hegde, M., Vaimberg, E.W., Donovan, K.F., et al. (2016). Optimized sgRNA design to maximize activity and minimize off-target effects of CRISPR-Cas9. *Nature Biotechnology* 34(2)**,** 184.

Kim, H.K., Min, S., Song, M., Jung, S., Choi, J.W., Kim, Y., et al. (2018). Deep learning improves prediction of CRISPR-Cpf1 guide RNA activity. *Nat Biotechnol* 36(3)**,** 239-241. doi: 10.1038/nbt.4061.

Wong, N., Liu, W., and Wang, X. (2015). WU-CRISPR: characteristics of functional guide RNAs for the CRISPR/Cas9 system. *Genome Biol* 16**,** 218. doi: 10.1186/s13059-015-0784-0.

Xu, H., Xiao, T., Chen, C.H., Li, W., Meyer, C.A., Wu, Q., et al. (2015). Sequence determinants of improved CRISPR sgRNA design. *Genome Research* 25(8)**,** 1147-1157.

**Supplementary Table 2.** The comprehensive list of learning-based gRNA activity design tools in our comparison study.

| **Name** | **Model** | **Methods** | **Features** | **Reference** |
| --- | --- | --- | --- | --- |
| CNN-SVR | Deep learning | CNN, SVR | Seq+Epi | - |
| CNN | Deep learning | CNN | Seq+Epi | - |
| Seq-deepCpf1 | Deep learning | CNN | Seq | (Kim et al., 2018) |
| DeepCRISPR | Deep learning | DCDNN auto-encoder | Seq+Epi | (Chuai et al., 2018) |
| sgRNA Designer | Machine learning | LR, L1, L2, RF, GBR, SVM, L1-LR,SVM-LR | Seq+SS | (Doench et al., 2016) |
| SSC | Machine learning | L1 | Seq | (Xu et al., 2015) |
| WU-CRISPR | Machine learning | SVM | Seq+SS | (Wong et al., 2015) |

Note: CNN, convolutional neural network; SVR, support vector regression; DCDNN, deep convolutionary denosing neural network; LR, linear regression; SVM, support vector machine; L1, L1-regularized linear regression; L2, L2-regularized linear regression; RF, random forest; GBR, Gradient-boosted regression; Seq+Epi, sequence features and epigenetic features; Seq+SS, sequence composition and secondary structures of gRNA.

**References**

Chuai, G., Ma, H., Yan, J., Chen, M., Hong, N., Xue, D., et al. (2018). DeepCRISPR: optimized CRISPR guide RNA design by deep learning. *Genome Biol* 19(1)**,** 80. doi: 10.1186/s13059-018-1459-4.

Doench, J.G., Fusi, N., Sullender, M., Hegde, M., Vaimberg, E.W., Donovan, K.F., et al. (2016). Optimized sgRNA design to maximize activity and minimize off-target effects of CRISPR-Cas9. *Nature Biotechnology* 34(2)**,** 184.

Kim, H.K., Min, S., Song, M., Jung, S., Choi, J.W., Kim, Y., et al. (2018). Deep learning improves prediction of CRISPR-Cpf1 guide RNA activity. *Nat Biotechnol* 36(3)**,** 239-241. doi: 10.1038/nbt.4061.

Wong, N., Liu, W., and Wang, X. (2015). WU-CRISPR: characteristics of functional guide RNAs for the CRISPR/Cas9 system. *Genome Biol* 16**,** 218. doi: 10.1186/s13059-015-0784-0.

Xu, H., Xiao, T., Chen, C.H., Li, W., Meyer, C.A., Wu, Q., et al. (2015). Sequence determinants of improved CRISPR sgRNA design. *Genome Research* 25(8)**,** 1147-1157.

**Supplementary Table 3.**Performance comparison of CNN-SVR and other prediction models on various testing cell line data.

| **Model** | **Total** | **HCT116** | **HEK293T** | **HELA** | **HL60** |
| --- | --- | --- | --- | --- | --- |
| 1. **Spearman correlation** | | | | | |
| CNN-SVR | **0.714** | **0.705** | 0.822 | **0.706** | **0.599** |
| DeepCRISPR | 0.601 | 0.654 | **0.874** | 0.501 | 0.262 |
| sgRNA Designer | 0.421 | 0.513 | 0.283 | 0.430 | 0.312 |
| SSC | 0.173 | 0.252 | -0.103 | 0.200 | 0.441 |
| WU-CRISPR | 0.149 | 0.215 | -0.041 | 0.159 | 0.268 |
| 1. **AUROC** | | | | | |
| CNN-SVR | **0.928** | **0.936** | **0.978** | **0.924** | **0.893** |
| DeepCRISPR | 0.857 | 0.874 | 0.961 | 0.782 | 0.739 |
| sgRNA Designer | 0.639 | 0.741 | 0.410 | 0.675 | 0.792 |
| SSC | 0.615 | 0.666 | 0.476 | 0.621 | 0.811 |
| WU-CRISPR | 0.579 | 0.621 | 0.495 | 0.598 | 0.588 |

Note: This corresponds to bar graph in Figure 2. The tables from top to bottom respectively record the Spearman correlation and AUROC of CNN-SVR and other prediction models.

**Supplementary Table 4.**Performance comparison of CNN-SVR and other prediction models on various testing cell line data with a leave-one-cell-out procedure.

| **Model** | **Average** | **HCT116** | **HEK293T** | **HELA** | **HL60** |
| --- | --- | --- | --- | --- | --- |
| 1. **Spearman correlation** | | | | | |
| CNN-SVR | **0.714** | 0.722 | **0.824** | **0.695** | **0.614** |
| DeepCRISPR | 0.406 | **0.761** | 0.069 | 0.544 | 0.250 |
| sgRNA Designer | 0.241 | 0.494 | -0.151 | 0.380 | 0.242 |
| SSC | 0.198 | 0.252 | -0.103 | 0.200 | 0.441 |
| WU-CRISPR | 0.150 | 0.215 | -0.041 | 0.159 | 0.268 |
| 1. **AUROC** | | | | | |
| CNN-SVR | **0.947** | **0.939** | **0.979** | **0.932** | **0.938** |
| DeepCRISPR | 0.722 | 0.919 | 0.506 | 0.820 | 0.643 |
| sgRNA Designer | 0.593 | 0.720 | 0.457 | 0.636 | 0.556 |
| SSC | 0.644 | 0.666 | 0.476 | 0.621 | 0.811 |
| WU-CRISPR | 0.576 | 0.621 | 0.495 | 0.598 | 0.588 |

Note: This corresponds to bar graph in Figure 3. The tables from top to bottom respectively record the Spearman correlation and AUROC of CNN-SVR and other prediction models.

**Supplementary Table 5.** Spearman correlation of different deep learning-based methods by considering gRNA sequence only and incorporating both gRNA sequence and epigenetic features.

| **Model** | **HCT116** | **HEK293T** | **HELA** | **HL60** | **Average** |
| --- | --- | --- | --- | --- | --- |
| 1. **Sequence-only** | | | | | |
| CNN-SVR | **0.710** | **0.816** | **0.687** | **0.570** | **0.696** |
| DeepCRISPR | 0.650 | 0.035 | 0.510 | 0.200 | 0.349 |
| Seq-deepCpf1 | 0.659 | 0.672 | 0.650 | 0.524 | 0.626 |
| 1. **Sequence composition and epigenetic features** | | | | | |
| CNN-SVR | 0.722 | **0.824** | **0.712** | **0.614** | **0.718** |
| DeepCRISPR | **0.761** | 0.069 | 0.544 | 0.250 | 0.406 |

Note: This corresponds to bar graph in Figure 4.

**Supplementary Table 6.**Performance comparison of CNN-SVR by considering gRNA sequence only and integrating both gRNA sequence and GC content.

| **Model** | **HCT116** | **HEK293T** | **HELA** | **HL60** | **Average** |
| --- | --- | --- | --- | --- | --- |
| 1. **Spearman correlation** | | | | | |
| Sequence only | 0.640 | **0.730** | 0.619 | 0.515 | 0.626 |
| Sequence + GC content | **0.645** | 0.716 | **0.656** | **0.608** | **0.656** |
| 1. **AUROC** | | | | | |
| Sequence only | 0.933 | 0.976 | 0.923 | **0.932** | 0.941 |
| Sequence + GC content | **0.938** | **0.978** | **0.926** | 0.928 | **0.943** |

**Methods to visualize the trained model**

In order to visualize the importance of mononucleotide and dinucleotide, we first constructed a specific sequence which only contains one nucleotide (out of 4 possible nucleotides) at position k (range from 1 to 23). The rest position in this sequence was set to 0. Note that for each constructed sequence, the four corresponding epigenetic sequences were generated in the similar way. Such sequence and its epigenetic features were fed in the trained network. Second, the network returned a value which indicates how likely the given sequence is an efficiency gRNA. This value indicates the importance of this nucleotide at position k. Third, the mentioned process was repeated to achieve the scores for all the constructed subsequences. We put four nucleotides (A, C, G and T) and their importance at all positions together in a heatmap to visualize their importance. The darker blue in the heatmap illustrated the nucleotide is more informative at the specific position.

Next, we explored the importance of the dimers. To this end, we generated a special sequence which only contains one dimer (out of 16 possible dimers) at position k (range from 1 to 22) as the way described above. Then the aforementioned procedure was repeated to get a score vector for all the constructed sequences by feeding all the sequences to the well-trained model. Similarly, we put all dimers (y-axis) and their importance at all positions (x-axis) together in a heatmap to visualize their importance (see Figure S6).
